# X-ray Absorption Spectroscopy Study of the Effect of Rh doping in Sr_2_IrO_4_

**DOI:** 10.1038/srep23856

**Published:** 2016-03-30

**Authors:** C. H. Sohn, Deok-Yong Cho, C.-T. Kuo, L. J. Sandilands, T. F. Qi, G. Cao, T. W. Noh

**Affiliations:** 1 Center for Correlated Electron Systems, Institute for Basic Science (IBS), Seoul 08826, Korea; 2 Department of Physics and Astronomy, Seoul National University, Seoul 08826, Korea; 3 IPIT & Department of Physics, Chonbuk National University, Jeonju 54896, Korea; 4 Center for Advanced Materials, Department of Physics and Astronomy, University of Kentucky, Lexington, Kentucky 40506, USA

## Abstract

We investigate the effect of Rh doping in Sr_2_IrO_4_ using X-ray absorption spectroscopy (XAS). We observed appearance of new electron-addition states with increasing Rh concentration (x in Sr_2_Ir_1−x_Rh_x_O_4_) in accordance with the concept of hole doping. The intensity of the hole-induced state is however weak, suggesting weakness of charge transfer (CT) effect and Mott insulating ground states. Also, Ir *J*_eff_ = 1/2 upper Hubbard band shifts to lower energy as x increases up to x = 0.23. Combined with optical spectroscopy, these results suggest a hybridisation-related mechanism, in which Rh doping can weaken the (Ir *J*_eff_ = 1/2)–(O 2*p*) orbital hybridisation in the in-planar Rh-O-Ir bond networks.

Novel phenomena in 5*d* transition metal oxides (TMOs) have been a central subject in condensed matter physics. The large spin-orbit (SO) coupling transforms the 5*d* electron states from crystal-field-driven orbital states to certain SO-entangled *J*_eff_ states (*J*_eff_ : effective total angular momentum)^1^,^2^. Consequently, a variety of exotic phases, including a relativistic Mott insulator^1^,^2^,^3^,^4^,^5^,^6^, a quantum spin liquid^7^,^8^,^9^,^10^,^11^, and a topological insulator^12^,^13^,^14^, are shown and expected in 5*d* TMOs. In the case of the most well-known 5*d* TMO, Sr_2_IrO_4_, even a superconductivity is expected because its crystal structure^15^, low energy Hamiltonian^16^,^17^, and magnetic excitation^18^,^19^ are similar to those of the mother compound of high *T*_*C*_ cuprates, La_2_CuO_4_. Indeed, recent angle-resolved photoemission spectroscopy studies showed the cuprate-like Fermi arc band and the *d*-wave gap in K doped Sr_2_IrO_4_^20^,^21^.

One of the important issues to be resolved for 5*d* TMOs is how electron/hole doping affects their ground states. It is because the doping behaviours will provide us the clues for understanding emergent phenomena including superconductivity. Studies on La_2−x_Sr_x_CuO_4_, for instance, manifested a charge transfer ground state where doped holes reside in the ligand O orbitals^22^,^23^. Such hole states in O 2*p* orbitals form the Zhang-Rice singlet state^24^, which has been known as the key component of high *T*_*C*_ superconductivity. Therefore, studies on carrier doping effects on 5*d* TMOs are necessary.

This article focuses on the effect of Rh doping in Sr_2_IrO_4_. Our Rh *L*-edge X-ray absorption spectroscopy (XAS) study revealed that the valence of Rh at low Rh concentrations is mainly +3 rather than +4, consistent to recent studies^25^,^26^. Therefore, Sr_2_Ir_1−x_Rh_x_O_4_ can be viewed as hole-doped iridates analogous to hole-doped cuprates. With O *K*-edge XAS, we particularly report two distinct features. One is that a new electron-addition state emerges near the chemical potential as a result of Rh doping. The intensity of the hole-induced states is, however, rather weak compared to the case of cuprates or nickelates. This suggests that the charge transfer (CT) effect is weak and doped holes mainly reside in the Ir 5*d* orbitals, being consistent with known Mott insulating ground state of Sr_2_IrO_4_. The other feature is that the 5*d* upper Hubbard band (UHB) hybridised with in-plane oxygen (O_*p*_) 2*p* orbitals undergoes a strong redshift whereas the same UHB but hybridised with apical oxygen (O_*a*_) 2*p* orbitals hardly does. We propose that Rh doping can decrease the hybridisation between Ir and O via charge reconstruction in O_*p*_ ions and/or via the less extended nature of Rh wave function. Optical spectroscopy study further showed that the onset energy of CT transition from O_*p*_ 2*p* to Ir 5*d* indeed decreases with increasing Rh concentration, supporting the interpretation of the XAS results.

## Results

**Figure 1:**
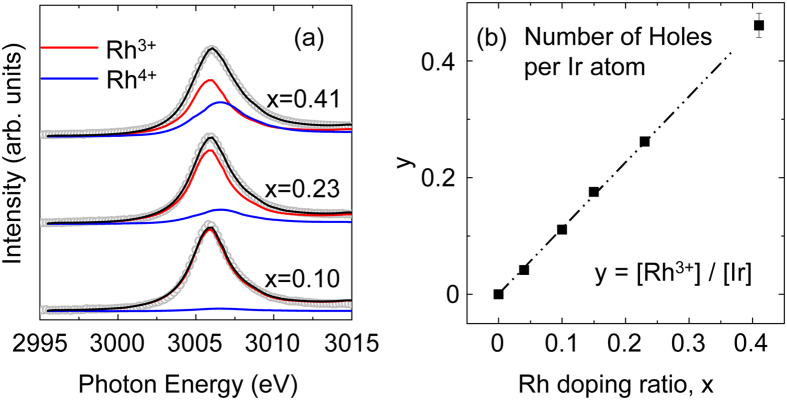
(**a**) Rh *L*_3_-edge XAS spectra of Sr_2_Ir_1−x_Rh_x_O_4_ crystals of x = 0.10, 0.23 and 0.41. The open circles show the experimental spectra and the black solid curves show the fitting results. The spectra of Rh^3+^ in Rh_2_O_3_ (red curve) and Rh^4+^ in Sr_2_RhO_4_ (blue curve) were taken from ref. 26 for the fitting. (**b**) The deduced values of Rh^3+^ concentration per one Ir site as a function of Rh doping ratio (x). Almost linear relation manifests the hole doping scheme at least for x ≤ 0.23. The error bars account for the uncertainty in determining the peak areas.

Rh *L*_3_-edge XAS was performed to estimate the hole concentration. Figure 1(a) shows the spectra of the Sr_2_Ir_1−x_Rh_x_O_4_ crystals for x = 0.10, 0.23 and 0.41. The peak near 3006 eV corresponds to the *L*_*3*_-edge white line, which represents the transitions from Rh 2*p*_3/2_ to 4*d* states. We fitted our spectra to linear combinations of known spectra of Rh^3+^ in Rh_2_O_3_ (the red lines) and Rh^4+^ in Sr_2_RhO_4_ (the blue lines)^26^. The valence of Rh is almost +3 rather than +4 in low-x samples (x ≤ 0.23) while the portion of Rh^4+^ ions starts to be prominent in x = 0.41 sample, consistent with a previous result^26^. Figure 1(b) shows the concentration ratio (y) of dopants to host metal ions (y = [Rh^3+^]/[Ir]), where [Rh^3+^] is the Rh^3+^ concentration according to our fitting results. It is clearly shown y increases almost linear to x, manifesting the hole doping effect.

**Figure 2:**
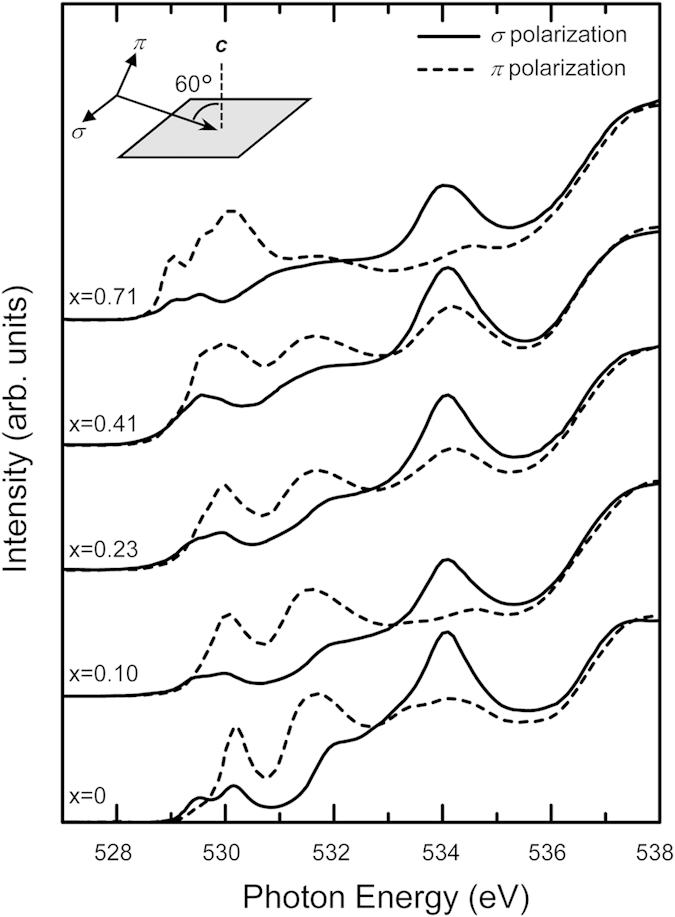
Polarization-dependent O *K*-edge XAS spectra of Sr_2_Ir_1−x_Rh_x_O_4_ crystals. The solid and dashed curves show the spectra taken with *σ*- and *π*-polarised light, respectively. The inset shows the measurement geometry.

The O *K*-edge XAS spectra show that the Rh doping considerably changes the electronic structure of Sr_2_IrO_4_ near the chemical potential. Figure 2 shows the O *K*-edge XAS spectra of Sr_2_Ir_1−x_Rh_x_O_4_ (x = 0, 0.10, 0.23, 0.41, and 0.71). The spectra near the threshold reflect transitions from O 1*s* core levels to unoccupied O 2*p* states hybridised with Ir/Rh *d* bands. The solid and dashed curves are the XAS spectra taken with *σ*- and *π*-polarised light, respectively. The experimental geometry is shown in the inset of Fig. 2. We fixed the angle of the incident beam to be 60°, so the polarisation of *σ*-polarised light was parallel to the sample surface, while the polarisation of *π*-polarised light was almost perpendicular to the surface. The huge polarisation dependence in the spectra suggests a quasi-2-dimensional nature in all the samples. With increasing Rh concentration, we observe large and consistent changes in the spectra, especially below the photon energy of 531 eV.

**Figure 3:**
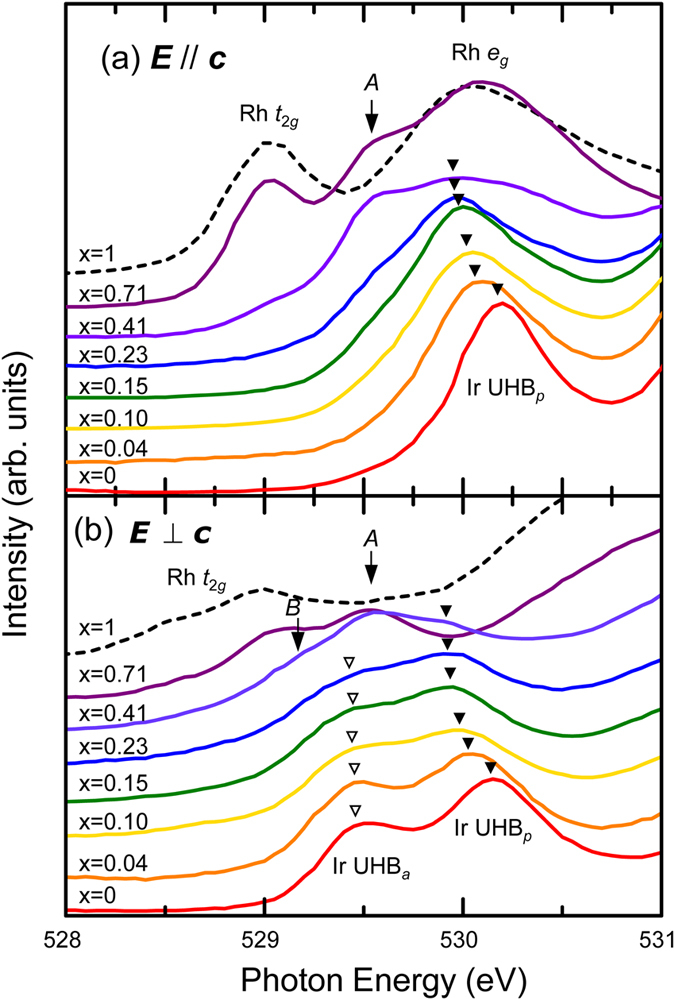
Low energy parts in the O *K*-edge XAS spectra with (**a**) ***E***//***c*** and (**b**) ***E***⊥***c***, where ***c*** is the surface normal axis. The spectra of Sr_2_RhO_4_ were taken from ref. 27. The ***E***⊥***c*** spectra are reproduced from the *σ*-polarisation data, and the ***E//c*** spectra are deduced from the relation: 
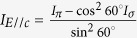
. Solid (open) triangles highlight the peak positions of the Ir *J*_eff_ = 1/2 UHB hybridised with the in-plane (apical) O 2*p*.

We can assign the peaks below 531 eV as in-plane and apical (O_*p*_ and O_*a*_) 2*p* states hybridised with Ir or Rh *d* orbitals, consistent with the previous studies^1^,^27^. Figure 3 shows the O *K*-edge XAS spectra of Sr_2_Ir_1−x_Rh_x_O_4_ near the threshold with the polarisation vectors (***E***) (a) parallel and (b) perpendicular to the normal axis of the surface (***c*** in the inset of Fig. 2), respectively. In the case of pure Sr_2_IrO_4_, there exist a single peak near 530.2 eV in the ***E***//***c*** spectrum and a doublet near 529.6 eV and 530.2 eV in the ***E***⊥***c*** spectrum. It is well known that the unoccupied state at lowest energy in Sr_2_IrO_4_ is close to a singlet of Ir 5*d J*_eff_ = 1/2 UHB, 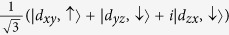. Thus, we attribute the split (of roughly 0.6 eV) as a chemical shift due to different chemistry between O_*p*_ and O_*a*_^1^,^27^. The peaks highlighted by filled triangles in Fig. 3 are the Ir 5*d* UHB hybridised with O_*p*_ 2*p* orbitals (UHB_*p*_). Likewise, the peaks marked as open triangles correspond to the Ir 5*d* UHB hybridised with O_*a*_ 2*p* orbitals (UHB_*a*_).

With increasing x, we observe appearance of lower-energy peaks, *A* and *B*, as highlighted in Fig. 3. The energies of peaks *A* and *B* are clearly distinct from those of the Sr_2_IrO_4_ and Sr_2_RhO_4_ peaks. Such lower-energy peaks are generally shown in hole-doped systems including nickelates^28^ or cuprates^22^,^23^, because new electron-addition states appear due to hole doping. Since the energy of such states should be close to that of original occupied states, the difference in energy between UHB and the hole-induced states should roughly reflects the charge gap. We see that the energy difference between peak A and Ir UHB_*p*_ (also, between peak *B* and Ir UHB_*a*_) is roughly 0.5 eV, similar to the known value of the charge gap in Sr_2_IrO_4_. Therefore, peaks *A* and *B* correspond to the electron-addition states hybridised with O_*p*_ and O_*a*_ orbitals, respectively. The energy difference between peaks *A* and *B* (~0.6 eV) in Fig. 3(b) reflects the difference in chemistry between O_*p*_ and O_*a*_, similar to the cases of UHB_*p*_ and UHB_*a*_.

Apart from the low-energy peaks (*A* and *B*), it is clearly shown in both Fig. 3(a,b) that the UHB_*p*_ peaks shift to lower energy with increasing x. The amount of the UHB_*p*_ redshift with respect to the case of undoped iridate is approximately 0.25 eV at x = 0.23. Interestingly, the redshift is much less predominant in the case of UHB_*a*_. Such an anisotropic peak shift in the two peaks is not easy to understand because they share identical Ir 5*d* orbitals (*J*_eff_ = 1/2 UHB). As will be discussed in the next section, the redshifts are the signatures of reduced O 2*p*–Ir 5*d* hybridisation strength upon the hole doping.

To conclude this section, we observe two distinct features due to Rh doping in the O *K*-edge spectra: One is the emergence of new electron-addition states (*A* and *B*) at low energy and the other is a concomitant redshift of Ir UHB_*p*_. These will be further analysed in Discussion.

## Discussion

**Figure 4:**
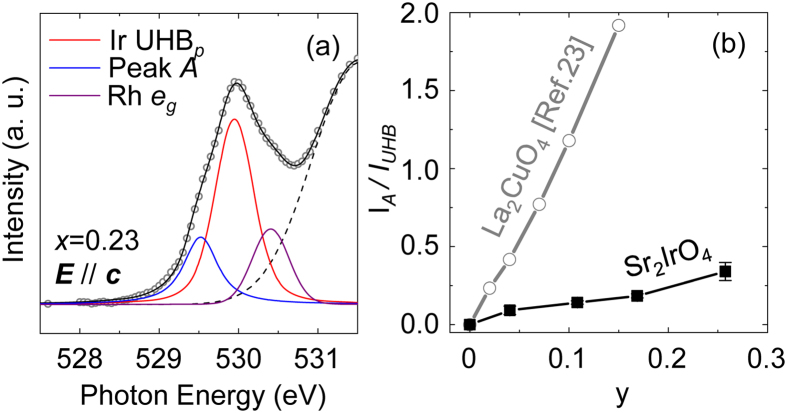
(**a**) The ***E***//***c*** spectra of x = 0.23 sample. The open circles are the experimental data and the black solid line is the fitting result. The red, blue, purple, and dashed curves are for Ir UHB_*p*_, peak *A*, Rh *e*_*g*_, and the Ir *e*_*g*_ background, respectively. (**b**) *I*_A_/*I*_UHB_ as functions of y (concentration ratio of dopants to host cations). The data of La_2_CuO_4_ is taken from ref. 23 for the comparison.

To describe the spectral evolution upon Rh doping quantitatively, we fitted the O *K*-edge spectra with ***E***//***c*** using Lorentz-Gaussian models. For x = 0.04–0.23 samples, only Rh^3+^ (*d*^6^) prevails so that there could exist three unoccupied states near the chemical potential: the hole-induced states (peak *A*), Ir UHB_*p*_, Rh *e*_*g*_ band with increasing order of energy. Figure 4(a) shows the best fitting result of x = 0.23 spectra using the three peaks with a higher energy background for Ir *e_g* band (dashed line). Meanwhile, for highly doped samples (x ≥ 0.41), it was difficult to obtain reliable fitting results because of the mixed contributions of Rh^3+^ and Rh^4+^ states.

We note that the intensity of peak *A* in Rh doped Sr_2_IrO_4_ is much smaller than those in cuprates and nickelates. Figure 4(b) shows the areal intensity of peak *A* normalised by that of UHB_*p*_, namely *I*_*A*_/*I*_UHB_ of the Rh-doped iridates as a function of y. Small error bars account for the uncertainty in determining the peak intensity. For comparison, we appended the *I*_*A*_/*I*_UHB_ values in (La_1−x_Sr_x_)_2_CuO_4_^23^ as a function of the number of holes per Cu as well. For the case of the cuprates, *I*_*A*_/*I*_UHB_ exceeds 1 even at y = 0.1, representatively showing the strong nature of CT insulator. In contrast, *I*_*A*_/*I*_UHB_ in Rh doped Sr_2_IrO_4_ at y = 0.11 (or x = 0.10), is only approximately 0.14. This indicates that the CT effect in iridates is much weaker compared to that in the cuprates. This observation is consistent with the Mott insulating ground state in Sr_2_IrO_4_, in which doped holes should reside mainly in metal *d* orbitals rather than ligand orbitals.

**Figure 5:**
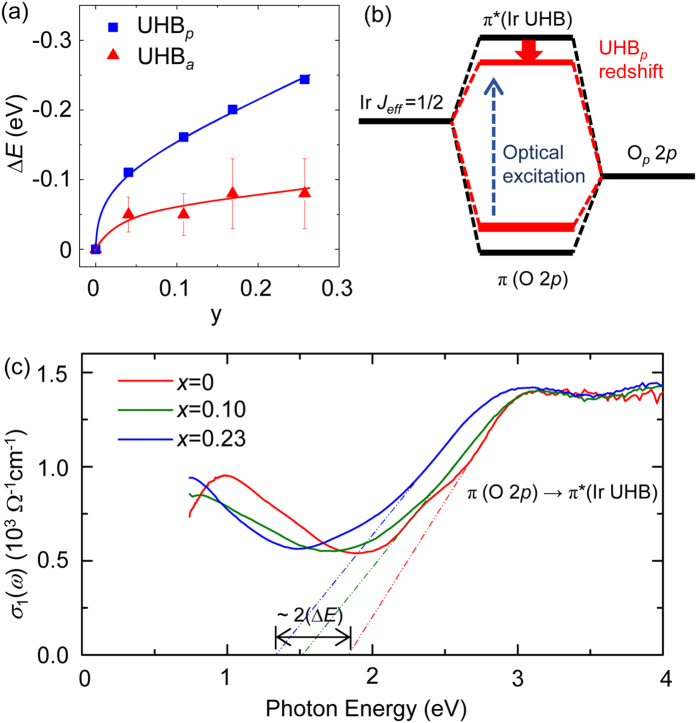
(**a**) Relative peak shifts (Δ*E*) of UHB_*p*_ (squares) and UHB_*a*_ (triangles) as functions of y with error bars. The solid lines are guides to the eye. (**b**) Schematic diagram of hybridisation change in Rh doped Sr_2_IrO_4_. (**c**) *σ*_1_(*ω*) obtained by optical spectroscopy. The extrapolations toward the abscissa roughly visualize the onsets of the charge transfer excitations from in-plane O 2*p* to Ir 5*d* UHB.

Another important finding in the O *K*-edge XAS spectra is that only UHB_*p*_ undergoes a redshift whereas UHB_*a*_ does not despite they represent the same UHB. Figure 5(a) shows the amount of redshifts (Δ*E*) of UHB_*p*_ and UHB_*a*_ with respect to those of undoped Sr_2_IrO_4_ as a function of y. Only the data for x ≤ 0.23, which exhibit little secondary phase (Rh^4+^), are collected. The error bars in the figure account for the uncertainty in determining the peak position through the fitting processes. In order to understand the origin of the redshifts, we first suspect the consistency with the structural evolution. It has been reported that with increasing x, the Ir-O bond is slightly shortened in average and the Ir-O-Ir bonding angle increases in average^29^. This moderate structural distortion would tend to increase the hybridisation strength of the Ir *J*_eff_ = 1/2 UHB–O 2*p* orbitals, which should have resulted in a blueshift of UHB_*p*_ or UHB_*a*_. Therefore, structural change upon Rh doping cannot explain the redshifts of UHB.

The plausible mechanism is the decrease of orbital hybridisation in the Ir-O bonds. The energy of UHB can be significantly influenced by the orbital hybridisation of bare Ir *J*_eff_ = 1/2 state and O 2*p* state. The schematic for the orbital hybridisation is illustrated in Fig. 5(b). As a result of the hybridisation, formed are an antibonding state π*, which is commonly called “Ir UHB”, above the chemical potential and a bonding state π, commonly called “O 2*p*”, below the chemical potential. What is observed in the O *K*-edge XAS data is the lowering of π*(UHB) hybridised with O_*p*_ (UHB_*p*_). Therefore, the redshift of UHB_*p*_ can be easily understood if Rh doping can effectively reduce the orbital hybridisation. This mechanism is more effective in the in-planar directions because the influence of Rh can reach Ir only via the in-plane O, namely, through Rh-O_*p*_-Ir bond chains due to quasi-2-dimensional crystal structure of Sr_2_IrO_4_. This explains why UHB_*p*_ suffers much more intense redshift compared to UHB_*a*_ (see Fig. 5(a)).

We propose two possible microscopic origins how Rh doping can decrease the orbital hybridisation between Ir *d* and O 2*p* orbitals. One is related to the difference of valence of ions. When the iridate is doped with Rh^3+^ ions, charge redistribution in O_*p*_ ions occurs: Since Rh can supply only 3 electrons to the O_*p*_ ions, O_*p*_ tend to attract more electrons from neighbouring Ir ions. It effectively increases the electronegativity of O_*p*_ in Ir-O_*p*_ bond, thereby making the Ir-O_*p*_ bond ionic. Similar mechanism but opposite trend has been discussed in H doped VO_2_^30^. The enhanced ionic character of the Ir-O_*p*_ bond can decrease hybridisation between two ions. The other is related to the less extended nature of Rh wave functions. According to the results of previous dynamical mean-field theory calculations, hybridisation between Ir and O ions is much stronger than that between Rh and O ions^31^. Because the valence *d* orbitals comprise the admixture of Ir and Rh *d* orbitals, Rh doping can effectively decrease the hybridisation between Ir 5*d* and O 2*p* orbitals.

To support the hybridisation mechanism discussed above, we measured the difference in energy between *π* and π* using optical spectroscopy. Figure 5(c) shows the in-plane real part of optical conductivity *σ*_1_(*ω*) of the x = 0, 0.10, and 0.23 samples. According to a previous study^32^, the broad features above photon energy of 2.5 eV can be attributed to a charge excitation from *π* (O 2*p*) to π* (Ir UHB). Meanwhile, the broad peak near 1 eV and the bump near 2.3 eV can be attributed to Ir *d-d* transition, which is less relevant to the main interest in this work. It is clearly observed that the onset energy of the high energy bump decreases with increasing x. This suggests that the Ir UHB–O 2*p* hybridisation becomes weaker as the hole doping is promoted, consistent with our XAS observations and interpretations.

The energy differences between *π* and π* bands can be roughly estimated from the extrapolations toward the abscissa. Compared to the case of undoped Sr_2_IrO_4_, the onset energy of x = 0.23 (x = 0.10) decreased by approximately 0.5 eV (0.3 eV), which is about two times larger than the amount of the UHB_*p*_ redshift (shown in Fig. 5(a)). This is consistent with the hybridisation mechanism for weakened hybridisation not only lowers the π* band but also lifts up the *π* band by similar magnitude. Therefore, *σ*_1_(*ω*) data supports the hybridisation mechanism for the UHB redshifts.

It is worthwhile to consider the consequence of reduced Ir UHB–O 2*p* hybridisation due to Rh doping on the low-energy physics in Sr_2_IrO_4_. It is well known that the low energy physics in strongly correlated electron systems can be largely determined by the ratio of on-site Coulomb interaction (*U*) to the single electron bandwidth (*W*). According to the results of a recent *ab initio* calculation, the value of *U* would decrease due to enhanced ligand screening^31^ so that *U*/*W* should decrease altering the electronic structure near the bandgap. However, the results of recent angle-resolved photoemission experiment show that Rh doping induces only a rigid shift of bands without an appreciable change in band dispersions^33^. This controversy can be resolved by noting that the value of *W* should decrease as well, because the decrease in hybridisation strength (by Rh doping) will reduce the bandwidth of the UHB. Therefore, the value of *U*/*W* should not change substantially.

In conclusion, Rh doped Sr_2_IrO_4_ exhibit peculiar evolution in electronic structure: i) a charge transfer state emerges near the chemical potential but the feature is not that strong as in the hole-doped cuprate, consistent to the Mott insulating ground state, and ii) the Ir UHB energy is lowered by a few tenths eV due to reduced Ir *J*_eff_ = 1/2 UHB–O 2*p* hybridisation strength upon the hole doping.

## Methods

### Materials

High-quality single crystals Sr_2_Ir_1−x_Rh_x_O_4_ (0 ≤ x ≤ 0.71) were synthesized from off-stoichiometric quantities of SrCl_2_, SrCO_3_, IrO_2_, and RhO_2_ using self-flux method. Detailed methods are described elsewhere^29^.

### X-ray absorption spectroscopy

Polarization-dependent O *K*-edge X-ray absorption spectroscopy (XAS) was performed at the 2A beamline of the Pohang Light Source in total electron yield mode. To obtain a clean surface, we cleaved samples *in situ* in an ultra-high vacuum (~6 × 10^−8^ Pa). We fixed the angle of the incident beam as 60 degrees, and changed the direction of polarisation to resolve the in- and out-of-plane O responses. Rh *L*-edge XAS was performed at the 16A1 beamline of the National Synchrotron Radiation Research Center in Taiwan in fluorescence yield mode. All the XAS spectra were collected at room temperature.

### Optical spectroscopy

We performed ellipsometry measurement to obtain the real part of optical conductivity between 0.74 and 4 eV at room temperature using a V-VASE ellipsometer (J. A. Woollam Co.).

## Additional Information

**How to cite this article**: Sohn, C. H. *et al.* X-ray Absorption Spectroscopy Study of the Effect of Rh doping in Sr_2_IrO_4_. *Sci. Rep.* **6**, 23856; doi: 10.1038/srep23856 (2016).

## References

[b1] Kim B. J. *et al.* Novel *J*_eff_ = 1/2 Mott State Induced by Relativistic Spin-Orbit Coupling in Sr_2_IrO_4_. Phys. Rev. Lett. 101, 076402 (2008). 18764560 10.1103/PhysRevLett.101.076402

[b2] Kim B. J. *et al.* Phase-Sensitive Observation of a Spin-Orbital Mott State in Sr_2_IrO_4_. Science 323, 1329–1332 (2009). 19265017 10.1126/science.1167106

[b3] Moon S. J. *et al.* Dimensionality-Controlled Insulator-Metal Transition and Correlated Metallic State in 5*d* Transition Metal Oxides Sr_n+1_Ir_n_O_3n+1_ (n = 1, 2, and ∞). Phys. Rev. Lett. 101, 226402 (2008). 19113493 10.1103/PhysRevLett.101.226402

[b4] Okabe H. *et al.* Ba_2_IrO_4_: A spin-orbit Mott insulating quasi-two-dimensional antiferromagnet. Phys. Rev. B 83, 155118 (2011).

[b5] Comin R. *et al.* Na_2_IrO_3_ as a Novel Relativistic Mott Insulator with a 340-meV Gap. Phys. Rev. Lett. 109, 266406 (2012). 23368593 10.1103/PhysRevLett.109.266406

[b6] Ohgushi K. *et al.* Resonant X-ray Diffraction Study of the Strongly Spin-Orbit-Coupled Mott Insulator CaIrO_3_. Phys. Rev. Lett. 110, 217212 (2013). 23745928 10.1103/PhysRevLett.110.217212

[b7] Chaloupka J., Jackeli G. & Khaliullin G. Kitaev-Heisenberg Model on a Honeycomb Lattice: Possible Exotic Phases in Iridium Oxides A_2_IrO_3_. Phys. Rev. Lett. 105, 027204 (2010). 20867736 10.1103/PhysRevLett.105.027204

[b8] Singh Y. *et al.* Relevance of the Heisenberg-Kitaev Model for the Honeycomb Lattice Iridates A_2_IrO_3_. Phys. Rev. Lett. 108, 127203 (2012). 22540620 10.1103/PhysRevLett.108.127203

[b9] Chaloupka J., Jackeli G. & Khaliullin G. Zigzag Magnetic Order in the Iridium Oxide Na_2_IrO_3_. Phys. Rev. Lett. 110, 097204 (2013). 23496744 10.1103/PhysRevLett.110.097204

[b10] Cao G. *et al.* Evolution of magnetism in the single-crystal honeycomb iridates (Na_1−x_Li_x_)_2_IrO_3_. Phys. Rev. B 88, 220414(R) (2013).

[b11] Takayama T. *et al.* Spin-orbit coupling induced semi-metallic state in the 1/3 hole doped hyper-kagome Na_3_Ir_3_O_8_. Sci. Rep. 4, 6818 (2014). 25351992 10.1038/srep06818 PMC4212231

[b12] Shitade A. *et al.* Quantum Spin Hall Effect in a Transition Metal Oxide Na_2_IrO_3_. Phys. Rev. Lett. 102, 256403 (2009). 19659103 10.1103/PhysRevLett.102.256403

[b13] Kim C. H., Kim H.-S., Jeong H., Jin H. & Yu J. Topological Quantum Phase Transition in 5*d* Transition Metal Oxide Na_2_IrO_3_. Phys. Rev. Lett. 108, 106401 (2012). 22463430 10.1103/PhysRevLett.108.106401

[b14] Kim H.-S., Kim C. H., Jeong H., Jin H. & Yu J. Strain-induced topological insulator phase and effective magnetic interactions in Li_2_IrO_3_. Phys. Rev. B 87, 165117 (2013).

[b15] Crawford M. K. *et al.* Structural and magnetic studies of Sr_2_IrO_4_. Phys. Rev. B 49, 9198–9201 (1994). 10.1103/physrevb.49.9198 10009706

[b16] Wang F. & Senthil T. Twisted Hubbard Model for Sr_2_IrO_4_: Magnetism and Possible High Temperature Superconductivity. Phys. Rev. Lett. 106, 136402 (2011). 21517402 10.1103/PhysRevLett.106.136402

[b17] Watanabe H., Shirakawa T. & Yunoki S. Monte Carlo Study of an Unconventional Superconducting Phase in Iridium Oxide *J*_eff_ = 1/2 Mott Insulators Induced by Carrier Doping. Phys. Rev. Lett. 110, 027002 (2013). 23383933 10.1103/PhysRevLett.110.027002

[b18] Kim J. *et al.* Magnetic Excitation Spectra of Sr_2_IrO_4_ Probed by Resonant Inelastic X-Ray Scattering: Establishing Links to Cuprate Superconductors. Phys. Rev. Lett. 108, 177003 (2012). 22680895 10.1103/PhysRevLett.108.177003

[b19] Kim J. *et al.* Excitonic quasiparticles in a spin–orbit Mott insulator. Nat. Commun. 5, 4453 (2014). 25029968 10.1038/ncomms5453

[b20] Kim Y. K. *et al.* Fermi arcs in a doped pseudospin-1/2 Heisenberg antiferromagnet. Science 345, 187–190 (2014). 24925913 10.1126/science.1251151

[b21] Kim Y. K., Sung N. H., Denlinger. J. D. & Kim B. J. Observation of a *d*-wave gap in electron-doped Sr_2_IrO_4_. Nat. Phys. 12, 37–41 (2016).

[b22] Nücker N., Fink J., Fuggle J. C., Durham P. J. & Temmerman W. M. Evidence for holes on oxygen sites in the high-*T*_*c*_ superconductors La_2−x_Sr_x_CuO_4_ and YBa_2_Cu_3_O_7−y_. Phys. Rev. B 37, 5158–5163 (1988). 10.1103/physrevb.37.5158 9943693

[b23] Chen C. T. *et al.* Electronic states in La_2−x_Sr_x_CuO_4+δ_ probed by soft-x-ray absorption. Phys. Rev. Lett. 66, 104–107 (1991). 10043153 10.1103/PhysRevLett.66.104

[b24] Zhang F. C. & Rice T. M. Effective Hamiltonian for the superconducting Cu oxides. Phys. Rev. B 37, 3759–3761 (1988). 10.1103/physrevb.37.3759 9944993

[b25] Klein Y. & Terasaki I. Insight on the electronic state of Sr_2_IrO_4_ revealed by cationic substitutions. J. Phys.: Condens. Matter 20, 295201 (2008).

[b26] Clancy J. P. *et al.* Dilute magnetism and spin-orbital percolation effects in Sr_2_Ir_1−x_Rh_x_O_4_. Phys. Rev. B 89, 054409 (2014).

[b27] Moon S. J. *et al.* Electronic structures of layered perovskite Sr_2_MO_4_ (M = Ru, Rh, and Ir). Phys. Rev. B 74, 113104 (2006).

[b28] Kuiper P., Kruisinga G., Ghijsen J., Sawatzky G. A. & Verweij H. Character of holes in Li_x_Ni_1−x_O and their magnetic behavior. Phys. Rev. Lett. 62, 221–224 (1989). 10039954 10.1103/PhysRevLett.62.221

[b29] Qi T. F. *et al.* Spin-orbit tuned metal-insulator transitions in single-crystal Sr_2_Ir_1−x_Rh_x_O_4_ (0 ≤ x ≤ 1). Phys. Rev. B 86, 125105 (2012).

[b30] Filinchuk Y. *et al.* *In situ* diffraction study of catalytic hydrogenation of VO_2_: Stable phases and origins of metallicity. J. Am. Chem. Soc. 136, 8100–8109 (2014). 24825186 10.1021/ja503360y

[b31] Sohn C. H. *et al.* Orbital-Dependent Polaron Formation in the Relativistic Mott Insulator Sr_2_IrO_4_. Phys. Rev. B 90, 041105(R) (2014).

[b32] Martins C., Aichhorn M., Vaugier L. & Biermann. S. Reduced effective spin-orbital degeneracy and spin-orbital ordering in paramagnetic transition-metal oxides: Sr_2_IrO_4_ versus Sr_2_RhO_4_. Phys. Rev. Lett. 107, 266404 (2011). 22243172 10.1103/PhysRevLett.107.266404

[b33] Cao Y. *et al.* Hallmarks of the Mott-Metal Crossover in the Hole Doped *J* = 1/2 Mott insulator Sr_2_IrO_4_. arXiv :1406.4978. 10.1038/ncomms11367 PMC4844699 27102065

